# Lifestyle Satisfaction Among Jazan University Employees: A Cross-Sectional Study Exploring Lifestyle Choices and Influencing Factors

**DOI:** 10.7759/cureus.55338

**Published:** 2024-03-01

**Authors:** Ibrahim M Gosadi, Anwar M Makeen, Mohammad A Jareebi, Mona H Elmahdy, Maged El-Setouhy, Sarah M Salih, Anas E Ahmed, Amani Abdelmola, Rafaa J Jafar, Yara A Mutaen

**Affiliations:** 1 Department of Family and Community Medicine, Faculty of Medicine, Jazan University, Jazan, SAU; 2 Department of Community Medicine, Faculty of Medicine, Benha University, Benha, EGY; 3 Department of Community, Environmental, and Occupational Medicine, Faculty of Medicine, Ain Shams University, Cairo, EGY; 4 Faculty of Medicine, Jazan University, Jazan, SAU

**Keywords:** satisfaction, jazan university, saudi arabia, employees, choices, lifestyle

## Abstract

Background: Lifestyle includes habits, behaviors, values, attitudes, and economic levels that define an individual or group's way of living for people living in the same region at a specific time. In the last few decades, with urbanization and modernization, most adults, especially in Arab countries, including Saudi Arabia, have adopted a sedentary, less active lifestyle. This study aims to assess lifestyle choices and satisfaction among employees of Jazan University, Jazan, Saudi Arabia.

Methods: This was a cross-sectional study that was conducted in the Jazan University campus in the southwestern region of Saudi Arabia. Data were collected through personal interviews conducted by trained medical students. A structured questionnaire was filled out during the interviews. Data analysis was conducted using R software (version 4.2.3) (R Development Core Team, Vienna, Austria).

Results: This study involved 1126 employees of Jazan University, with a response rate of 75%. The occupational distribution was as follows: 576 (51%) in administrative positions, 516 (46%) as faculty members, and 34 (3%) as healthcare workers. In terms of physical activity, 488 (43%) engaged in less than 150 minutes of weekly physical activity, while 363 (32%) reported no physical activity at all. Regarding body weight satisfaction, 590 (52%) were satisfied, while 536 (48%) were not. Males reported a higher satisfaction in body weight, physical activity, and eating behavior. Dietary choices, such as eating fruits and vegetables, low-fat meats, and avoiding high-sugar foods, positively correlated with satisfaction in eating behavior and body weight. The assessment of satisfaction with body weight, physical activity level, and eating behavior indicates that some university affiliates are satisfied with their lifestyle despite having unhealthy lifestyle choices.

Conclusion: The current findings indicate that Jazan University affiliates are experiencing a high prevalence of unhealthy lifestyles, especially in terms of low levels of physical activity, selection of unhealthy food items, and overweight and obesity. This study should be followed up by interventional designs to investigated best evidence-based approaches for lifestyle behavior change, especially among aging populations such as university affiliates.

## Introduction

Lifestyle includes habits, behaviors, values, attitudes, and economic levels that define an individual or group's way of living. It usually describes the characteristics of people living in the same region at a specific time. It can also be considered individually to define personal behavior and functions in life, including activities and dietary habits [1]. This lifestyle significantly impacts human health. The American Heart Association considers a healthy lifestyle as "eating right and being active, getting enough sleep, practicing mindfulness, managing stress, keeping mind and body fit, connecting socially, and more" [2].

In the last few decades, with urbanization and modernization, most adults, especially in Arab countries, including Saudi Arabia, have adopted a sedentary, less active lifestyle [3-6]. However, a recent systematic review in Arab countries showed significant variation in physical activity among adults, ranging from 34.2% to 96.9%. The authors attributed these differences to the use of different methodologies in research across these countries [7]. In addition to the unhealthy lifestyle, changes in eating behavior have been supported by the proliferation of fast food chains in Arab countries, especially in Saudi Arabia. These changes are often associated with numerous non-communicable diseases, including obesity, hypertension, ischemic heart diseases, and diabetes mellitus [8-9]. Hence, it is recommended for adults to engage in at least 150 minutes of moderate physical activity per week [10-11].

For all the aforementioned reasons, the government program the Vision of Saudi Arabia 2030 aims to promote healthy lifestyles, including increased public engagement in sports and physical activity, and to strengthen prevention against threats to health, such as non-communicable diseases and physical inactivity [12]. Furthermore, there is a particular focus on the concept of life satisfaction and quality of life in relation to a healthy lifestyle as a necessity in one's life [13]. A study conducted in 27 European countries showed a positive effect of a healthy BMI on life satisfaction [14]. Other studies explored the role of delayed gratification in relation to lifestyle and life satisfaction [15], as well as satisfaction among seniors and special groups [16-18]. Although some publications in Arab countries have discussed life satisfaction, they have not explored its relation to lifestyle [19].

As lifestyle behaviors and choices impact individuals' health, well-being, and life satisfaction, it is essential to evaluate the health status within academic institutions. Currently, there is limited evidence concerning lifestyle choices among residents of the Jazan region. In a study that targeted a sample of 234 primary healthcare physicians in Jazan, it was concluded that the majority of the physicians were exhibiting high levels of overweight and obesity, low physical activity, and imbalanced eating behavior [20]. Similarly, in another study that targeted 244 adults from Jazan upon their visits to primary healthcare centers to assess their risk of dyslipidemia development, it was reported that physical inactivity was the most frequently reported risk factor followed by the consumption of food high in fat contents [21]. Nonetheless, it must be noted that these findings are acquired from studies with small sample sizes and limited follow-up, larger scale studies are needed to study lifestyle choices among adults in the region. 

The importance of the assessment of lifestyle choices among adults, especially among those who are aging, is related to the increased risk of the development of chronic diseases or its impact on chronic disease control. The assessment of lifestyle risk factors and the tendency to adopt a modification toward a healthy lifestyle is a cornerstone in any primary, secondary, and tertiary preventive measures for chronic diseases. One of the theories that explain the phases of changing behavior is the transtheoretical model (stages of change) [22]. The model is composed of six stages, namely, pre-contemplation, contemplation, preparation, action, and maintenance, and it has been indicated to be suitable for the prevention of chronic diseases [23]. 

It is possible to argue that adults who are satisfied with their poor lifestyle choices are considered within the pre-contemplation stage, and are less likely to adopt a healthy lifestyle change. This signifies the importance of assessing lifestyle among adults and assessing whether they are satisfied with their lifestyle or not. This is necessary to decide the nature of health modification intervention, where those who are at the pre-contemplation stage might require a different intervention in comparison to those at the contemplation or preparation stage.

There is a gap in knowledge concerning whether adults are satisfied with their lifestyle choices or not, especially in the Saudi Arabian context. In the current investigation, affiliates of an academic institution were selected to study lifestyle choices and satisfaction with the choices to investigate lifestyle as an important health determinant among adults. Jazan University is the only university in the southern western region of Saudi Arabia and it is possible to argue that its employees represent an appropriate sample of the adult population with different backgrounds and demographic characteristics. This might facilitate the assessment of lifestyle choices and employees' satisfaction with their lifestyle and may initiate a basis for broader community-based assessment. This study aims to assess lifestyle satisfaction among Jazan University employees. The findings can provide valuable information to help policymakers determine the healthcare priorities of Jazan University employees and enable them to effectively enhance the adoption of healthy lifestyle strategies among them.

## Materials and methods

Study context

This study represents a phase of a project aiming to conduct a health needs assessment of students, teaching staff, and administrative staff affiliated with Jazan University. A cross-sectional design was selected and the recruitment was performed between May and June 2023. The project received ethical approval from the Standing Committee for Scientific Research of Jazan University (IRB approval number REC-44/10/630 dated May 3, 2023). Participation in the assessment was voluntary and anonymous and was conducted after obtaining informed consent from the participants.

Data collection tool

The data collection tool for conducting the health needs assessment was obtained after consulting relevant international literature associated with the development of community health needs assessment tools in European [24] and American contexts [25]. The contents of the assessment tool were reviewed by a panel of experts in family and community medicine to evaluate its comprehensiveness and suitability for assessing health needs among university affiliates. The developed health needs assessment tool measured several components, including the demographics of the participants, lifestyle determinants of health, and participants' satisfaction with their lifestyles.

The lifestyle determinants measured in the current study were adopted from the Saudi Guidelines for the Prevention and Management of Obesity [26]. The participants were asked about their reported body weight and height, which enabled the estimation of the participants' BMIs. The current level of physical activity was assessed by asking the participants whether they adhered to the clinical recommendation of performing 150 minutes or more of exercise per week or not. The assessment of eating behavior was done by asking the participants about the five elements eat-well plate, which assesses the consumption of whole-grain products, fruits and vegetables, low-fat meats, low-fat products, and whether foods high in sugar (such as snacks) are avoided. Satisfaction with the reported body weight, physical activity, and eating behavior was assessed by asking the participants about their degree of satisfaction via one assessment item for each parameter. Responses about satisfaction were measured using a Likert scale varying between 1 (indicating the lowest level of satisfaction) and 10 (indicating the highest level of satisfaction) with body weight, physical activity, or eating behavior. Cronbach's coefficient alpha was estimated for the items assessing satisfaction with lifestyle choices and produced a reasonable internal validity of 0.77.

Data collection process

Data collection was conducted through personal interviews facilitated by trained medical students, with the structured questionnaire filled out during the interview. The identification and approaching of university employees took place in person within their natural working settings on the university campus. Those who consented to participate were interviewed, thus completing the recruitment process. This study targeted university employees, and any identified individuals who were not working at the university at the time of the data collection were excluded.

Convenience sampling was used to recruit university employees when approached in their work settings. University campuses are separated in Saudi Arabia according to gender. Additionally, this study involved university employees who belong to different employment categories. To ensure equal representation of various employees according to their gender and employment characteristics, equal recruitment was ensured to recruit similar proportions of the university employees according to their employment category, faculty, and gender. Initially, data collectors were divided into groups to ensure equal recruitment of participants from both male and female campuses, and they were further divided to recruit university employees from all faculties and from different employment categories.

Jazan University has nearly 5,000 employees. A sample of 1,500 university employees was estimated to be recruited for the current investigation. Sample size estimation was based on the sample size formula for a cross-sectional study design: \(N= (z^2^p (1 - p))/d^2^\), where N is the initial sample size, p is the anticipated population proportion under focus, z^2^ is the confidence level (usually 1.96 for a 95% confidence interval), and d is the absolute precision required. Using this equation and substituting the following parameters: p = 50% (as the survey targeted multiple health indicators, it is safer to use 50%, which provides the largest sample size), a 95% confidence interval, and an error margin of no more than 2%, the initial sample size was calculated to be 1,200 participants. The sample size was further increased by 25% to account for the non-response rate, resulting in a final sample size of 1,500 participants.

Data analysis

Data analysis was conducted using R software (version 4.2.3) (R Development Core Team, Vienna, Austria). The risk factors under examination fall into two distinct categories. The first category comprises socio-demographic characteristics, including variables such as age, gender, education, residence, income, nationality, languages spoken, employment status, social standing, and housing. The second category focuses on health-related parameters, including measurements of height, weight, BMI, physical activity levels, smoking habits, khat usage, and dietary behaviors. Smoking and khat usage habits were classified according to the reported practice at the time of recruitment where the participants were classified as ex-smokers or khat ex-users regardless of the period since the cessation. The outcome variables of interest pertain to the satisfaction levels regarding weight, physical activity, and dietary behaviors among the study participants.

Our analysis began with an overview of the sample characteristics. Qualitative variables were depicted using frequencies and proportions, while quantitative variables were summarized with mean values and their corresponding standard deviations (SDs). The utilized Likert scales to measure the satisfaction of the employees about their body weight, physical activity, and eating behavior were grouped based on the median values of the satisfaction level of each measured parameter. Participants with satisfaction values of less than the median were classified as experiencing lower satisfaction levels, while those with a value equal to or more than the median were classified as having higher satisfaction levels. This categorization enabled the employment of multiple logistic regression analyses to explore the adjusted relationships between these outcome variables and the identified risk factors. To quantify these associations, we used odds ratios (ORs). Statistical significance was determined with a threshold of a p-value below 0.05.

## Results

A total of 1,500 participants were approached in the current investigation, of whom 1,126 participants agreed to participate, representing a response rate of 75%. Table 1 describes the socio-demographic characteristics of 1,126 employees of Jazan University in Saudi Arabia. The mean age of participants was 40 years (± 7.3), with 523 (46%) males and 603 (54%) females. Educational backgrounds varied, with 146 (13%) having high school or less, 468 (41.5%) having a bachelor’s degree, and 512 (45.5%) having postgraduate qualifications. In terms of residence, 297 (26%) lived in rural areas and 829 (74%) in urban areas. Incomes showed variation, with 105 (9%) earning less than 5,000 Saudi riyals, 401 (36%) falling in the 5,000-9,999 bracket, 374 (33%) earning 10,000-14,999, and 246 (22%) earning ≥15,000. The majority of participants were Saudi nationals, accounting for 792 (70%), followed by 118 Indians (10%), 101 Egyptians (9%), 68 Sudanese (6%), and other nationalities accounting for only 47 (5%) of the sample. Arabic was the primary language for 965 (85.7%) participants, with 161 (14.2%) reporting the use of other languages. The occupational distribution was 576 (51%) in administrative positions, 516 (46%) as faculty members, and 34 (3%) as healthcare workers. Regarding marital status, 188 (17%) were single, 865 (77%) were married, and 73 (6%) were divorced or widowed. Regarding housing, most participants, 463 (41.1%), indicated that they resided in rented accommodations, followed by 278 (24.7%) who reported living in owned villas, 231 (20.5%) in owned apartments, and 154 (13.7%) in owned traditional houses.

**Table 1:** Socio-demographic characteristics of 1,126 employees from Jazan University, Saudi Arabia SD: Standard deviation, n: Sample size

Characteristics	Mean ± SD
Age	40 ± 7.3 years
Characteristics	Frequency (%)
Gender	
Male	523 (46%)
Female	603 (54%)
Education	
High school and below	146 (13%)
Bachelor's degree	468 (41.5%)
Postgraduate	512 (45.5%)
Residence	
Rural	297 (26%)
Urban	829 (74%)
Income (Saudi Riyal)	
Less than 5000	105 (9%)
5000-9999	401 (36%)
10000-14999	374 (33%)
≥15000	246 (22%)
Nationality	
Saudi	792 (70%)
Indian	118 (10%)
Egyptian	101 (9%)
Sudanese	68 (6%)
Other	47 (5%)
Languages	
Arabic	965 (85.7%)
Other languages	161 (14.2%)
Employment	
Administrative	576 (51%)
Healthcare workers	34 (3%)
Faculty members	516 (46%)
Social status	
Single	188 (17%)
Married	865 (77%)
Divorced/widowed/widower	73 (6%)
Housing	
Owned apartment	231 (20.5%)
Owned traditional	154 (13.7%)
Owned Villa	278 (24.7%)
Rented	463 (41.1%)

Table 2 provides insights into the lifestyles and satisfaction levels of 1,126 employees at Jazan University in Saudi Arabia. The average height in this cohort was 164 cm, with a weight of 72 kg, resulting in an average BMI of 27. When considering BMI categories, 402 (36%) fell into the normal weight range, while 460 (41%) were overweight, and 243 (22%) were classified as obese. In terms of physical activity, 488 (43%) engaged in at least 150 minutes of weekly physical activity, while 363 (32%) reported no physical activity at all. As for smoking behavior, the majority, 879 (78%), had never smoked, while 132 (12%) were current smokers. Khat chewing was relatively uncommon, with 1,045 (93%) reporting never doing it. Regarding eating habits, 674 (60%) consumed whole-grain products, 352 (31%) managed to eat at least five servings of fruits and vegetables daily, and 646 (57%) opted for low-fat meats like fish. Nearly half, 514 (46%), avoided high-sugar foods, and 468 (42%) chose low-fat products. Regarding body weight satisfaction, 590 (52%) reported higher satisfaction, while 536 (48%) reported lower satisfaction. Similarly, 537 (47.7%) expressed higher satisfaction with their physical activity, and 638 (57%) exhibited higher satisfaction levels with their eating habits.

**Table 2:** BMI, lifestyle choices, and satisfaction with lifestyle among 1,126 employees from Jazan University, Saudi Arabia SD: Standard deviation

Characteristics	Mean ± SD
Height	164 ± 9.1 cm
Weight	72 ± 15 kg
BMI	27 ± 4.9
Characteristics	Frequency (%)
BMI categories	
Underweight	21 (2%)
Normal weight	402 (36%)
Overweight	460 (41%)
Obese	243 (22%)
Physical activity	
No physical activity	363 (32%)
Less than 150 minutes of weekly physical activity	488 (43%)
150 minutes or more of weekly physical activity	275 (24%)
Smoking behavior	
Never	879 (78%)
Ex-smoker	54 (5%)
Current	132 (12%)
Passive	61 (5%)
Khat chewing	
Never	1045 (93%)
Ex-user	46 (4%)
Current	35 (3%)
Eating behavior	
Consumption of whole-grain products (such as whole-grain bread and brown rice)	674 (60%)
Consuming a minimum of five servings of fruits and vegetables per day	352 (31%)
Choosing low-fat meats (such as fish)	646 (57%)
Avoiding foods high in sugar (such as snacks)	514 (46%)
Choosing low-fat products	468 (42%)
Body weight satisfaction
Higher satisfaction	590 (52%)
Lower satisfaction	536 (48%)
Physical activity satisfaction	
Higher satisfaction	537 (47.7%)
Lower satisfaction	589 (52.3%)
Eating behavior satisfaction	
Higher satisfaction	638 (57%)
Lower satisfaction	488 (43%)

The findings presented in Table 3 demonstrate how different predictors influence satisfaction levels related to body weight, physical activity, and eating behavior among 1,126 employees at Jazan University in Saudi Arabia. Factors associated with increased satisfaction levels include being male (OR = 1.66, 95% CI: 1.22-2.27, p = 0.001 for body weight satisfaction; OR = 1.81, 95% CI: 1.30-2.54, p < 0.001 for physical activity satisfaction; OR = 2.21, 95% CI: 1.61-3.04, p < 0.001 for eating behavior satisfaction), using languages other than Arabic (OR = 2.74, 95% CI: 1.31-5.97, p = 0.009 for body weight satisfaction), being a current khat user (OR = 2.42, 95% CI: 1.02-5.82, p = 0.046 for physical activity satisfaction), engaging in moderate physical activity (OR = 7.25, 95% CI: 5.12-10.43, p < 0.001 for physical activity satisfaction), engaging in vigorous physical activity (OR = 19.92, 95% CI: 13.10-30.87, p < 0.001 for physical activity satisfaction, OR = 1.56, 95% CI: 1.08-2.25, p = 0.017 for eating behavior satisfaction), eating fruits and vegetables daily (OR = 1.61, 95% CI: 1.21-2.15, p < 0.001 for eating behavior satisfaction), choosing low-fat meats (such as fish) (OR = 1.47, 95% CI: 1.12-1.94, p = 0.005 for body weight satisfaction, OR = 1.65, 95% CI: 1.27-2.16, p < 0.001 for eating behavior satisfaction), avoiding high-sugar foods (OR = 1.48, 95% CI: 1.13-1.95, p = 0.004 for eating behavior satisfaction), and using low-fat products (OR = 1.70, 95% CI: 1.29-2.25, p < 0.001 for eating behavior satisfaction).

**Table 3:** Associations between body weight satisfaction, physical activity satisfaction, and eating behavior satisfaction according to BMI and lifestyle choices among 1,126 employees from Jazan University, Saudi Arabia OR: Odds ratio; CI: Confidence interval; SR: Saudi riyal P<0.05 is considered significant

	Body weight satisfaction			Physical activity satisfaction			Eating behaviour satisfaction		
Predictors	OR	95% CI	p	OR	95% CI	p	OR	95% CI	p
Age	0.99	0.97 – 1.01	0.312	1.01	0.99 – 1.03	0.407	1.01	0.99 – 1.04	0.276
Gender (reference: female)									
Male	1.66	1.22 – 2.27	0.001	1.81	1.30 – 2.54	<0.001	2.21	1.61 – 3.04	<0.001
Nationality (reference: Saudi)									
Egyptian	0.80	0.42 – 1.49	0.475	1.26	0.64 – 2.47	0.497	1.00	0.53 – 1.88	0.990
Indian	0.53	0.21 – 1.31	0.177	0.90	0.35 – 2.26	0.816	0.92	0.35 – 2.34	0.863
Sudanese	0.69	0.35 – 1.38	0.301	0.95	0.45 – 1.99	0.896	0.77	0.39 – 1.54	0.462
Other	0.42	0.18 – 0.95	0.038	0.63	0.26 – 1.50	0.295	0.70	0.31 – 1.62	0.402
Language (reference: Arabic language)									
Other Languages	2.74	1.31 – 5.97	0.009	1.16	0.55 – 2.50	0.695	1.59	0.75 – 3.50	0.233
Education (reference: high school and below)									
Postgraduate	0.78	0.39 – 1.54	0.470	0.65	0.32 – 1.34	0.244	0.57	0.29 – 1.12	0.105
Bachelor's degree]	0.99	0.62 – 1.58	0.953	0.72	0.44 – 1.19	0.199	0.58	0.36 – 0.92	0.023
Employment (reference: faculty members)									
Administrative	0.54	0.31 – 0.95	0.033	0.95	0.53 – 1.70	0.871	0.77	0.44 – 1.34	0.355
Healthcare worker	0.60	0.26 – 1.38	0.226	1.07	0.45 – 2.62	0.874	1.20	0.52 – 2.83	0.669
Social status (reference: single)									
Divorced/widow/widower	1.18	0.64 – 2.18	0.590	0.67	0.35 – 1.28	0.223	1.17	0.63 – 2.17	0.610
Married	1.25	0.85 – 1.83	0.257	1.04	0.69 – 1.55	0.864	1.31	0.89 – 1.91	0.166
Income (reference: less than 5000 SR)									
5000 to 9999 SR	0.75	0.46 – 1.23	0.258	0.95	0.57 – 1.59	0.850	0.80	0.49 – 1.30	0.369
10000-14999 SR	1.03	0.61 – 1.73	0.921	1.04	0.61 – 1.80	0.877	0.86	0.51 – 1.44	0.563
15000 SR and above	0.76	0.41 – 1.40	0.380	1.01	0.53 – 1.92	0.980	0.68	0.37 – 1.25	0.220
Residence (reference: rural)									
Urban	1.17	0.84 – 1.63	0.342	0.88	0.62 – 1.24	0.458	1.26	0.91 – 1.76	0.169
Housing (reference: rented)									
Owned apartment	1.01	0.67 – 1.52	0.952	1.05	0.68 – 1.63	0.817	0.74	0.49 – 1.12	0.157
Owned traditional house	1.59	0.96 – 2.65	0.073	1.30	0.76 – 2.23	0.340	1.16	0.70 – 1.93	0.574
Owned villa	1.19	0.79 – 1.79	0.409	0.94	0.61 – 1.45	0.780	0.78	0.52 – 1.17	0.231
BMI	0.84	0.81 – 0.87	<0.001	0.93	0.87 – 0.98	0.043	0.92	0.86 – 0.98	0.008
BMI category (reference: normal weight)									
Underweight	0.13	0.05 – 0.36	<0.001	0.88	0.30 – 2.72	0.827	0.36	0.13 – 1.02	0.055
Overweight	0.63	0.40 – 0.99	0.046	0.95	0.61 – 1.49	0.820	0.84	0.55 – 1.29	0.415
Obese	1.08	0.47 – 2.54	0.852	0.88	0.38 – 2.04	0.763	0.77	0.35 – 1.69	0.510
Smoking (reference: never)									
Current smoker	1.26	0.79 – 2.04	0.337	0.56	0.34 – 0.92	0.024	0.92	0.57 – 1.49	0.730
Ex-smoker	0.57	0.30 – 1.07	0.081	0.46	0.23 – 0.90	0.024	0.82	0.43 – 1.59	0.557
Passive smoker	1.02	0.57 – 1.84	0.945	0.92	0.49 – 1.73	0.792	0.97	0.53 – 1.76	0.908
Khat chewing (reference: never)									
Current	0.82	0.37 – 1.83	0.622	2.42	1.02 – 5.82	0.046	0.97	0.43 – 2.24	0.940
Ex-user	1.17	0.58 – 2.41	0.659	1.00	0.49 – 2.06	0.999	0.86	0.43 – 1.73	0.665
Physical activity (reference: no physical activity)									
A total of 150 minutes or more of weekly physical activity	0.97	0.71 – 1.31	0.827	7.25	5.12 – 10.43	<0.001	1.32	0.97 – 1.79	0.079
More than 150 minutes of weekly physical activity	1.37	0.96 – 1.95	0.086	19.92	13.10 – 30.87	<0.001	1.56	1.08 – 2.25	0.017
Eating behavior (reference: no): consumption of whole-grain products									
Yes	0.92	0.70 – 1.21	0.553	1.10	0.83 – 1.46	0.524	0.80	0.61 – 1.05	0.113
Eating behavior: consuming a minimum of five servings of fruits and vegetables per day									
Yes	1.23	0.92 – 1.64	0.161	1.08	0.80 – 1.46	0.599	1.61	1.21 – 2.15	0.001
Eating behavior: choosing low-fat meats (such as fish)									
Yes	1.47	1.12 – 1.94	0.005	1.23	0.93 – 1.64	0.150	1.65	1.27 – 2.16	<0.001
Eating behavior: avoiding foods high in sugar									
Yes	1.09	0.83 – 1.43	0.553	0.95	0.71 – 1.26	0.715	1.48	1.13 – 1.95	0.004
Eating behavior: choosing low-fat products									
Yes	0.95	0.72 – 1.26	0.732	1.17	0.87 – 1.57	0.291	1.70	1.29 – 2.25	<0.001

Conversely, an increase of one SD in BMI was associated with lower satisfaction levels for all study outcomes (OR = 0.84, 95% CI: 0.81-0.87, p < 0.001 for body weight satisfaction, OR = 0.93, 95% CI: 0.87-0.98, p = 0.043 for physical activity satisfaction, OR = 0.92, 95% CI: 0.86-0.98, p = 0.008 for eating behavior satisfaction). Similarly, being underweight and overweight was associated with lower body weight satisfaction compared to normal-weight individuals (OR = 0.13, 95% CI: 0.05-0.36, p < 0.001 and OR = 0.63, 95% CI: 0.40-0.99, p = 0.046, respectively). Compared to Saudi nationality, other nationalities were associated with decreased physical activity satisfaction (OR = 0.42, 95% CI: 0.18-0.95, p = 0.038). Additionally, holding a bachelor’s degree was associated with decreased levels of eating behavior satisfaction (OR = 0.58, 95% CI: 0.36-0.92, p = 0.023). Being an administrative staff member was associated with decreased body weight satisfaction (OR = 0.54, 95% CI: 0.31-0.95, p = 0.033). Finally, current and ex-smokers were associated with decreased physical activity satisfaction compared to people who had never been smokers (OR = 0.56, 95% CI: 0.34-0.92, p = 0.024 and OR = 0.46, 95% CI: 0.23-0.90, p = 0.024, respectively). Factors that were not significantly associated with satisfaction levels include age, social status, income, residence, housing, and consumption of whole-grain products.

## Discussion

The current investigation aimed to assess the lifestyles of employees working at a Saudi university and to assess the magnitude of their satisfaction with their lifestyle. The findings indicate that only 36% reported having a normal BMI, and 63% of the recruited employees were either overweight or obese. When the participants were asked about their physical activity levels, only 24% reported adherence to the recommended level of physical activity, and 32% reported not performing any type of physical activity. Around 17% of the participants reported currently being smokers or having a past history of smoking. The assessment of the eating behavior concerning the recommended guidelines for food consumption indicated that the least adopted recommendation was concerning the consumption of fruits and vegetables (31%), followed by the consumption of low-fat products (42%).

Although 64% of the participants reported having abnormal BMI, 52% of the employees reported being satisfied with their body weight, which indicates that some employees are satisfied with their body weights despite their abnormality. Similarly, although only 24% of the participants reported optimum adherence to the recommended level of physical activity, nearly half of the sample (48%) reported currently having higher levels of satisfaction with their physical activity level. Finally, nearly 70% of the sample did not meet the recommended guidelines for proper eating behavior, yet more than half exhibited higher satisfaction levels with their eating habit. These findings suggest that some university employees can be classified as satisfied with their lifestyle choices even when they are classified as having unhealthy lifestyles. Assessment of factors associated with the level of body weight, eating behavior, and physical activity satisfaction indicate that gender, nationality, spoken language, employment, BMI levels, smoking history, khat chewing, and physical activity level are factors associated with satisfaction level of the measured parameters.

The findings of the current study can be compared to similar national and international investigations. In a similar study by Gosadi et al. that targeted primary healthcare physicians in the Jazan region in 2019 to assess their lifestyle choices, it was noted that nearly 70% of the recruited 234 physicians were either overweight or obese, which is slightly higher than the prevalence of overweight and obesity in the current sample (63%). Similarly, 27% of the physicians reported no engagement in physical activity, which is similar to the findings of the current investigation (32%). Finally, Gosadi et al. indicated that the consumption of fruits and vegetables among physicians varied between 42% and 56%, which is higher than the current findings (31%). The study by Gosadi et al. did not measure the satisfaction of the physicians concerning their lifestyle choices. Nonetheless, it was indicated that poor lifestyle choices among physicians may affect the proper provision of lifestyle counseling to the targeted community [20]. 

The high prevalence of overweight and obesity, limited physical activity, and improper eating behavior detected in our recent investigation is similar to previous assessments conducted in Saudi Arabia. In a review that assessed risk factors for metabolic syndrome in Saudi Arabia, it was concluded that Saudis within various age groups consume high levels of fast food and food high in salt content, exhibit low consumption of fruits and vegetables, and are physically inactive [27]. The World Health Survey (Saudi Arabia 2019) reported an increasing prevalence of diabetes, hypertension, obesity, and dyslipidemia among Saudis in recent years, which can be partially explained by lifestyle risk factors [28]. Screening for diabetes, hypertension, and dyslipidemia was emphasized as a preventive measure against the expected rise of these chronic conditions in the upcoming years [29]. 

Smoking history characteristics of the current sample indicated that 12% were current smokers, while 5% were previous smokers. These findings are similar to the findings of the study by Mahfouz et al. which indicated that 15% of 736 healthcare workers from Jazan were smokers, while 11.5% were ex-smokers [30]. Mahfouz et al. indicated the public health implications of the identified prevalence of smoking among healthcare workers since these workers are less likely to contribute to tobacco control efforts in the targeted community. It is possible to argue that smoking among university professors might pose a harmful effect on their students since university professors can be viewed as role models by their students.

The current study showed that some university affiliates reported adopting some unhealthy lifestyle choices, yet reported higher satisfaction levels with their choices. This indicates that some university affiliates might be at the pre-contemplation phase of stages of change and are less likely to intend to take action in the future to adopt a healthy lifestyle. Similarly, those who reported having unhealthy lifestyle choices and reported lower satisfaction levels with their choices can be considered in the contemplation phase. There is currently limited evidence concerning lifestyle choice satisfaction among adults and how the satisfaction is likely to impose a change. For example, a protocol for systematic review was identified assessing the utilization of the transtheoretical model on lifestyle changes in older persons [31], which indicates that the scientific community is considering assessing the application of the model to enhance the current understanding of how lifestyle change can be presumed as a healthcare challenge, especially among older population, such as university affiliates.

In a study by Rojo-Ramos et al. that assessed physical activity satisfaction among 545 physical education students from Spain, it was concluded that the majority of students were satisfied with their physical activity level [32]. However, no demographic factors were identified to be associated with the level of physical activity satisfaction, which is different from the current findings suggesting that physical activity level can be associated with gender, smoking and khat chewing behavior, and physical activity level. Studies that assessed eating behavior satisfaction are currently limited. A study that assessed eating behavior among 430 to identify predictors of food satisfaction indicated that increased enjoyment of food, food responsiveness, and hunger were detected to increase eating satisfaction [33]. Nonetheless, these findings are not comparable to the findings of the current investigation where certain demographic factors were identified to be associated with the level of eating behavior satisfaction.

The findings of the current investigation indicate that some university affiliates are satisfied with their body weight despite having an abnormal body weight. In a Spanish study that involved a sample of 1081 adults to assess their body weight satisfaction, it was concluded that women were less satisfied with their body weight [34], which is similar to the findings of the current study. In another Spanish study that assessed the influence of excess weight, body image and satisfaction, self-stigma, positivity, and happiness among 100 adults who were overweight or obese, it was concluded that individuals who are overweight or obese with elevated body satisfaction and positivity are less likely to report being happy [35]. Although the current study did not measure body image, stigma, positivity, and happiness, it is possible to argue that the current findings suggest that some university affiliates are happy with their body weight and reported being satisfied incurring the need for more focused intervention to initiate adoption of a behavior change toward body weight management.

Limitations

The current study has several areas of strengths and weaknesses. The main strength of the current study is related to its ability to reach a representative sample of the university affiliates and the ability to provide a sample with good variability in demographic factors. Furthermore, the data collection was performed via interviews on-site on the university campus, and thus, is more likely to enhance the reporting quality of the collected data. The study's main weakness was the potential for inaccuracies in the self-reported body weights and heights of the participants. Additionally, other confounding factors may have influenced the lifestyle satisfaction levels of the employees, which can be an area for future investigations. Finally, though an effort was made to recruit a representative sample according to the university employee characteristics, it is not possible to neglect the potential influence of non-probability sampling on the ability to generalize the findings of the study to similar communities. Nonetheless, it is possible to argue that the study was able to reach a good representation of university affiliates according to their employment categories and gender.

## Conclusions

The current findings indicate that Jazan University affiliates are experiencing a high prevalence of unhealthy lifestyles, especially when considering low levels of physical activity, selection of unhealthy food items, and raised prevalence of overweight and obesity. The assessment of satisfaction with body weight, physical activity level, and eating behavior indicates that some university affiliates can be presumed as satisfied with their lifestyles despite having unhealthy lifestyle choices. This calls for the adoption of specific interventions designed to target the university affiliates according to the presumed stage of health behavior change to enhance university affiliates' well-being and subsequently organizational effectiveness. Furthermore, this study should be followed up by interventional designs to investigate the best evidence-based approaches for lifestyle behavior change, especially among aging populations such as university affiliates. 
